# The Role of Parental Involvement in Youth Sport Experience: Perceived and Desired Behavior by Male Soccer Players

**DOI:** 10.3390/ijerph18168698

**Published:** 2021-08-17

**Authors:** Valerio Bonavolontà, Stefania Cataldi, Francesca Latino, Roberto Carvutto, Michele De Candia, Gioacchino Mastrorilli, Giulia Messina, Antonino Patti, Francesco Fischetti

**Affiliations:** 1 Department of Basic Medical Sciences, Neuroscience and Sense Organs, School of Medicine, University of Bari “Aldo Moro”, 70124 Bari, Italy; valerio.bonavolonta@uniba.it (V.B.); stefania.cataldi@uniba.it (S.C.); roberto.carvutto@uniba.it (R.C.); michele.decandia@uniba.it (M.D.C.); sceva94@hotmail.it (G.M.); francesco.fischetti@uniba.it (F.F.); 2 Sport and Exercise Sciences Research Unit, Department of Psychology, Educational Sciences and Human Movement, University of Palermo, 90133 Palermo, Italy; giuliamessina00@gmail.com (G.M.); antonino.patti01@unipa.it (A.P.)

**Keywords:** parental involvement, youth sport experience, directive behavior, parenting educational style, pressure

## Abstract

Parents play a key role in the youth sports educational experience. They are responsible for the introduction of their children to physical or sporting education and their involvement has been associated with sport participation in early stages. The aims of this cross-sectional study were, first, to assess the perceived and desired parental involvement by children and, secondly, to examine their satisfaction or dissatisfaction with any specific behavior. 80 male soccer players filled the Parental Involvement in Sport Questionnaire (PISQ) before or after a training session in presence of a coach. PISQ results revealed excessive active involvement and pressure, insufficient praise and understanding and satisfactory directive behavior from children’s parents. Our findings suggest that excessive parental involvement can cause pressure on children who would prefer parental participation characterized by praise and understanding. A balance between a supporting involvement without putting too much pressure is needed by the parents. To prevent burnout and dropout and to facilitate future practice, parents should be counseled (possibly by a sport educator) on how to positively support their children concerning their sport experience.

## 1. Introduction

As it has been strongly emphasized during the last decade, a physically active lifestyle benefits health and social domains [1,2]. Indeed, general well-being and good individual bio-psycho-social functioning are affected by physical inactivity [3] which is a risk factor for illnesses in adulthood [4]. Participation in sports in childhood and adolescence is related to an active lifestyle practice in young adulthood [5,6] and has also been reported to increase the probability of a high amount of physical activity in later life [1,7].

Parents play an important role in this participation, as they usually contribute to children’s initial sport involvement and provide concrete and emotional support throughout children’s sport careers [8]. Moreover, parents are those responsible to introduce their children to physical and sporting activity (PSA) [9] providing transport, access [10], educational, and emotional and economic support. Indeed, parents bring their children to the competition venue and remain there, which means that parents can potentially affect the child and their behavior with several instances [11].

The nature of parent involvement in organized youth sport has often been debated and criticized [12], with both positive and negative implications to children’s experience. Thus, the role of parents’ involvement in this educational process needs to be better investigated and clarified. Parent involvement consists of both parent support and pressure behaviors which make it a complex and multidimensional construct [13,14,15,16,17,18,19,20]. However, some studies supported the opposite [21,22,23,24]. Indeed, several studies reported that children appreciate the participation and interest of parents in monitoring their sport educative activities, but that parents must be alert and aware of the level and manner of their engagement so that the experience of their children in the sport context can be positive [25,26,27,28,29,30,31,32].

Parent support has been linked to several factors related to sports participation such as child enjoyment and enthusiasm, autonomy, and self-perception of sport skill [15]. Parent pressure, instead, has been linked to negative outcomes related to sport performance such as the perceptions of a threatening environment, discontent, anxiety and negative impact [16,19]. Indeed, in players perceiving more pressure from their parents, a positive association with amotivation and a negative one with enjoyment was found [22].

All the aforementioned aspects together with parents’ physical activity contribute to define their attitudes and behaviors regarding the sport experience of their children [29,31,32]. Parental involvement and the potential pressure on children’s sport educational process are then crucial and necessary to examine, as these aspects might condition children’s efforts in those activities [25,26].

Some researchers reported negative aspects besides the positive influence of parental support. Indeed, studies showed that parental expectations are a source of stress among young athletes, possibly due to the awareness of their parents’ efforts [33,34].

Hellstedt [35] proposed a model that describes three styles of involvement: underinvolved, moderately involved, and overinvolved. Underinvolved families, then parents, show little to no interest in the child’s sport, talent, or progress. Moderately involved athlete families balance firm parental direction with the child’s power to make her or his own decisions about goals, participation, and commitment. Overinvolved parents are emotionally involved with the child’s sport experiences and performance, and they tend to project their lives into their child’s sport successes [35]. Parental behaviors include dreams of fame, considering their child’s sport experience as an investment for the future, and also invading the coaches’ field of action, attending practices constantly and focusing on winning rather than on child’s skill and motor development, enjoyment, and health.

Similarly, Lee and MacLean [36] have defined active involvement referring to children who consider their parents actively involved in their sport experience, a typology of parental behavior that cause positive reaction and satisfaction in young athletes [24]. Directive behavior [36], instead, is when children feel controlled by their parents in sports promoting the perception of pressure [24]. Parental pressure has been defined as parental behaviors that symbolize high or even unattainable expectations in the minds of child athletes [17].

Moreover, a narrative review by Amado et al. [37] reported that parental pressure towards children’s sport was positively related with stress, while it was negatively associated with enjoyment and motivation [25]. Conversely, a parental participation characterized by praise and understanding [36] favor increased levels of players’ enjoyment and motivation for sport [22].

Although parental involvement has been related to sport participation in early ages, there is still poor research about this topic in youth sport education. In line with previous studies, it is predictable that excessive parental involvement could be related to excessive pressure among young athletes. Therefore, the objective of the present study was twofold: to better clarify parental involvement in youth sport experience and the perceived support and pressure by children. The first aim was to assess perceived and desired parental involvement by children; the second aim was to analyze children’s satisfaction or dissatisfaction with specific behaviors by their parents comparing perceived and desired behaviors.

## 2. Materials and Methods

### 2.1. Study Design

The present work is an analytical cross-sectional study that collected data through a psychological scale, the Parental Involvement Sport Questionnaire (PISQ) [36]. In addition, discrepancies between perceived and desired behavior ratings versus satisfaction value were compared. Any possible correlations between the variables existed was also analyzed.

### 2.2. Participants and Procedures

Eighty male soccer players aged 11–14 (M age = 12.5, SD = 1.1) years were recruited from a local recreational soccer sport club (U.S.D. Modugno Invictus LAM, Puglia, Italy) and volunteered to participate in the study. Participants had at least three years of practice experience and they all took part in the corresponding youth competitions. Participants and their families were informed about the purpose of the study and informed consent was obtained by parents. Then, children self-completed a questionnaire before or after a regular training session, in the presence of both their coach and a research team member. All questionnaire data were collected and treated in agreement with the ethical guidelines provided by the American Psychological Association. PISQ was used to measure how children perceived the parental involvement and how they desired their parents to be involved in their sport activity [36]. In the past, large research supported the cross-cultural validity of PISQ [23,24,38,39] that has previously been used with Italian sample [38] (Danioni et al., 2017). PISQ comprises 19 items and uses a 5-point Likert scale (from 1 = never to 5 = always) asking children about which frequency each described behavior was exhibited by parents and also desired by the children. The scale provides three scores that assess the exhibited behavior of the parents: (1) active involvement (AI; five items, e.g., “Do your parents take an active role in running your club?”), (2) praise and understanding (PU; four items, e.g., “After a contest do your parents praise you for trying hard?”), and (3) directive behavior (DB; ten items, e.g., “Before a contest do your parents tell you how to do your competition?”). There is also a single item “Do your parents put pressure on you concerning your sport?” that aim to assess exhibited parental pressure (Pr). In addition, the same subscales assesses children desired behavior by their parents: (1) active involvement (AI; five items, e.g., “Would you like your parents to discuss about your progress with your coach?”), (2) praise and understanding (PU; four items, e.g., “Would you like your parents to show they understand how you are feeling about your sport?”), and (3) directive behavior (DB; ten items, e.g., “Before a contest, would you like your parents to tell you how to compete?”). Finally, the item “Would you like your parents to put pressure on you concerning your sport?” was used to assess desired parental pressure (PR). Children’s satisfaction or dissatisfaction with any specific behavior was calculated by discrepancies between scores of perceived and desired behaviors: Discrepancy = Perceived Behavior—Desired Behavior. The range of possible discrepancies goes from −4 to +4.

### 2.3. Statistical Analysis

All analyses were performed using SPSS Statistics Version 23.0(IBM Corp, Armonk, NY, USA) and the data were presented as group mean values and standard deviations. Cronbach’s alpha was calculated to assess the internal consistency of the psychological measures; according to Cohen [40] (2011) scores from 0.70 to 0.79 are considered reliable, from 0.80 to 0.90 as highly reliable, and >0.90 as very highly reliable. Normality of all variables was assessed using the Shapiro–Wilk test. To compare discrepancy scores with satisfaction value (i.e., zero), a single sample *t*-test was applied. When a positive or negative difference was observed a two-tailed test was performed. Effect size (d) for the one-sample *t*-test was calculated according to Cohen’s definition of small, moderate and large (a value equal to 0.20, 0.50, and 0.80, respectively) [41]. In addition, Pearson’s correlation coefficient (r) was calculated to measure the direction and strength of the relation between all the subscales: an r value of 0.5 to 0.7 is considered low, 0.7 to 0.8 is moderate, and 0.9 or above is good [42] (Vincent and Weir, 2012). Statistical significance was set at *p* ≤ 0.05.

## 3. Results

A post-hoc sample power (G * Power 3.1) indicated that given an α = 0.05, an effect size d = 0.5, our sample would allow a power = 0.996. Means, ranges and standard deviations of the four patterns collected by PISQ are reported in Table 1. Cronbach’s alpha for all subscales showed good to very good internal consistency as shown in Table 1. Significant differences from 0, considered as satisfaction value (recurrence rate 34.6%), indicated children’s satisfaction or dissatisfaction with any specific behavior.

**Figure 1:**
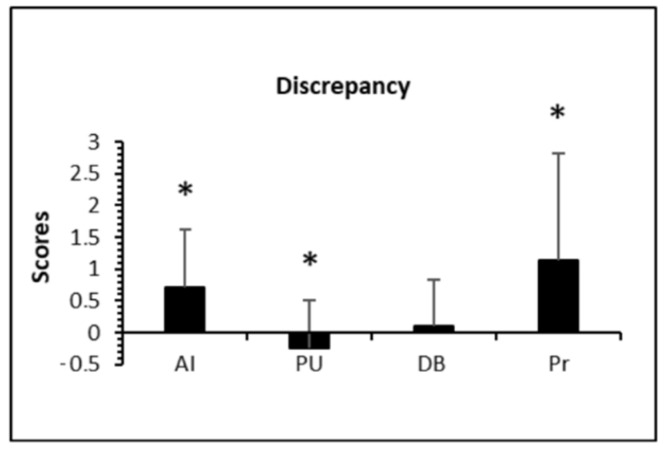
Discrepancies between ratings of perceived and desired behavior. Discrepancy = Perceived − Desired Behavior. Range of possible discrepancies goes from −4 to +4. Satisfaction value = 0. AI, active involvement; PU, praise and understanding; DB, directive behavior; Pr, pressure. * Significant discrepancy (*p* < 0.01).

**Figure 2:**
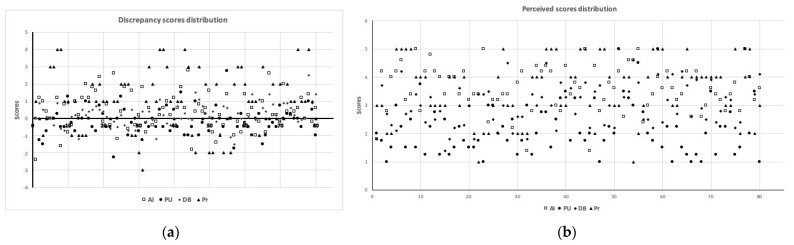
(**a**) Distribution of the sample discrepancy scores; (**b**) Distribution of the sample perceived scores. AI, active involvement; PU, praise and understanding; DB, directive behavior; Pr, pressure.

Single *t*-tests for Discrepancy scores revealed excessive active involvement (x = 0.72, t(79) = 6.95, *p* < 0.001, d = 0.78), insufficient praise and understanding (x = −0.25, t(79) = −2.98, *p* < 0.01, d = 0.33) but satisfactory directive behavior (x = 0.11, t(79) = 1.37, *p* = 0.175, d = 0.15) reported by children from their parents. The only item concerning levels of pressure indicated a similar discrepancy between perceived and desired parental behavior (x = 1.13, t(79) = 5.94, *p* < 0.001, d = 0.66). Discrepancy scores are shown in Figure 1 while in Figure 2 are shown the distribution of discrepancy scores (panel a) and the perceived scores (panel b), respectively.

In addition, bivariate Pearson correlations (r) results showed that desired active involvement was positively correlated both with desired pressure (r = 0.34, *p* < 0.01) and with pressure discrepancy (r = 0.24, *p* < 0.05).

## 4. Discussion

Parental involvement in the sport educational process of children is a controversial and multidimensional topic that needs clarification in terms of parental support, understanding, and pressure. The present data provide some indications that confirm the complexity of parent-child relationships and the importance of parenting educational style in youngsters’ sports activities, confirming that parents’ role can range from highly positive to highly negative in the youth sports experience.

Indeed, results indicate that children perceived moderate to high levels of parental involvement and pressure, moderate levels of directive behavior, and low levels praise and understanding. These results are not in agreement with previous findings [43] that reported that amateur hockey players aged 13–15 perceived more praise and understanding than active involvement or directive behavior from their parents. On the contrary, results indicate that children desire lower levels of parental involvement, directive behavior and pressure, while desiring higher levels of praise and understanding, possibly due to the social influence of soccer.

Regarding the levels of satisfaction, our findings showed excessive active involvement, insufficient praise and understanding, but satisfactory directive behavior on the part of parents; this result is not in line with Marsh et al. [44] who reported that active involvement and praise and understanding were positively correlated in club level swimmers and volleyball, soccer, and hockey players. Moreover, young athletes referred excessive levels of parent pressure, which is potentially related to excessive parental involvement. Results confirmed this relation as the correlation analysis found that desired active involvement was positively related both with desired pressure and the pressure discrepancy. This could be interpretated as children desiring lower levels of parental involvement and, thus, lower parent pressure.

The present findings are in line with Sánchez-Miguel et al. [22], but not with other studies [39,44] where all of the athletes desired more parental pressure but, in specializing years, they desired more parental praise and understanding. Parental involvement is perceived differently by the athletes in each athletic development phase, and it can become more salient over the years [45,46,47]. As the present study was conducted on a relatively homogeneous sample, although representative, future research may compare different developmental phases also through longitudinal studies. Moreover, as stated by Giannitsopoulou et al. [39], there might be differences in perceived involvement and pressure of parents by the young athletes depending on specific cultures. Another possible explanation for the results differing from previous studies, could be represented by the perception that soccer has in Italy, being the most popular sport compared to other countries.

The present findings are not in line with Lee and MacLean [36] who reported that directive behavior is the critical variable in the perception of parental pressure among young swimmers. Indeed, children showed satisfaction with their parents’ directive behavior, whereas the parents’ pressure was related to the excessive active involvement.

This study’s findings show that parental involvement, in some forms, can be negatively perceived by young athletes. Some studies attribute this phenomenon to subjective perceptions [48,49]. Thus, some athletes may perceive support from their parents as enjoyable and intrinsically motivating [50] while others may perceive such support as proper of high-performance participation, and thus as pressuring [21].

For this reason, parents should be aware and told that their behaviors may be analyzed and misinterpreted, thus resulting in negative outcomes that can contribute to lower the levels of motivation and to the loss of enthusiasm towards sport participation; young athletes may thus experience stress and conflicts with their parents, until burnout or dropout [24,25,28,34,51].

It could be then argued that a balance between a supporting involvement without putting too much pressure is needed by the parents. However, at the same time, there seems to be a fine line between being supportive and being overinvolved in children’s sporting participation depending on the parenting styles [52,53]. Holt [54] refers to youth sport parenting style as a complex dominion that needs to be sensitive to a wide range of perceptions and behaviors rather than to single variables. The same author also reported a reciprocal influence of children on their parents’ parenting styles.

Referring to enjoyment and sport participation, Gobbi et al. [55] reported that activities eliciting enjoyment or positive outcomes could instigate repeated or habitual participation in that activity and that football has been reported as an enriched environment that can increase enjoyment [56]. Therefore, if higher parental pressure causes increased level of stress in children and adolescents, this can compromise their future participation in PSA, favoring dropout and inactivity. Moreover, in a previous work by the same authors, it was concluded that perceived parental support seemed to be a key element in increasing children physical activity level [57].

It is then advisable that parents would be educated through a specific counseling that can help them to support and participate in the sporting experience of their children positively and in a less-invasive way, as reported by Sacks et al. [58]. Genevois [11] proposed a useful and simple technique by giving parents an observation sheet to help them in maintaining a certain level of neutrality in terms of body language while still being able to encourage their children verbally at key moments.

Coaches, and more effectively, sport educators, can create a motivational and educational positive climate that may help parents to be supportive without putting pressure. This win-win partnership could also help in the prevention of burnout and dropout. Indeed, recently, Morano et al. [59] reported that monitoring functional psycho-biosocial states, as consequences of environmental motivational aspects, can have a significant effect on contrasting burnout symptoms.

Moreover, in order to develop an effective strategy for a positive parental involvement, it is advisable an approach that would combine and integrate the action of sport educators with sport psychologists in non-formal contexts (such as recreational teams), where most of sport activities have place. In this perspective, it should be underlined the importance of the presence of a sport educator (i.e., a teacher), possibly by reconceptualizing the figure of the coach who must have several fundamental interrelated competences [60,61].

The present study has also some limitations that need to be considered: first, because of the study design, results need to be confirmed by multiple assessments across time to investigate how and if parental involvement affects or may vary across different periods of the season. Second, the sample could be increased taking into consideration more than one club from different locations, more sports, and also including female players. Further studies should also consider the role of national organizations (sports clubs, teaching institutions, National Associations Rulebooks for different ages) as important contributors that can influence parents’ behaviors [62]. Furthermore, as results are based only on children’s reports, future studies should consider parental responses. Finally, any differences between mother and father should also be verified. Indeed, Bloemhoff et al. [63] found that athletes desired more active involvement and more praise and understanding from their parents, especially from their fathers. Similarly, Stein [13] reported that child athletes enjoyed the most in their sport when their fathers were moderately involved, whereas the most stress was experienced when their mother’s involvement was perceived too little or too much.

## 5. Conclusions

The present study contributes to the understanding of parent involvement in sport educational process, suggesting that excessive parental involvement may be perceived as a source of pressure among young athletes. In addition, children seem to prefer a parental participation characterized by praise and understanding.

Further studies are needed concerning parental influences on children’s physical and sport activities experience to improve existing and future intervention programs and children and adolescent’s compliance to these activities.

## Figures and Tables

**Table 1:** Perceived and desired parental involvement in sports activity by children. Scores are presented as mean, range (1–5), and standard deviation (SD).

Score	Active Involvement *		Praise and Understanding ^†^		Directive Behavior		Pressure *	
	Perceived	Desired	Perceived	Desired	Perceived	Desired	Perceived	Desired
Mean	3.59	2.87	2.20	2.46	3.05	2.94	3.58	2.45
Range	1.4–5.0	1.2–5.0	1.0–5.0	1.0–5.0	1.4–5.0	1.4–5.0	1.0–5.0	1.0–5.0
SD	0.80	0.88	0.96	0.86	0.80	0.88	1.08	1.21
Cronbach α	0.72	0.74	0.74	0.75	0.88	0.90	0.92	0.91

* Excessive behavior: significant difference from satisfaction value (*p* < 0.05). ^†^ Insufficient behavior: significant difference from satisfaction value (*p* < 0.05). Satisfaction value: perceived—desired = 0.

## Data Availability

Dataset will be made available upon request.
